# The impact of body image disturbances on social avoidance in burn patients

**DOI:** 10.3389/fpsyt.2025.1714860

**Published:** 2025-12-08

**Authors:** Qingjiang Huang, Zhujing Li, Fengwen Yue, Weitian Shi, Ying Lei, Ting Jiang

**Affiliations:** Burn Plastic and Aesthetic Surgery, Beijing Anzhen Nanchong Hospital of Capital Medical University & Nanchong Central Hospital, Nanchong, Sichuan, China

**Keywords:** burn patients, body image disturbances, social avoidance, family support, psychological resilience

## Abstract

**Background:**

Body image serves as an auxiliary to nonverbal communication, facilitating the transmission of nonverbal information in interpersonal interactions. However, burn patients often experience body image disturbances due to scarring, skin pigmentation changes, and functional impairments, leading to communication difficulties and social anxiety. Regrettably, this phenomenon has received limited in-depth exploration in previous research.

**Methods:**

To address this gap, this study, based on the fear-avoidance model, randomly recruited 286 patients to analyze the impact of body image disturbances on social avoidance and its internal mechanisms.

**Results:**

The experimental results demonstrated that burn patients with higher levels of body image disturbances exhibited stronger tendencies toward social avoidance. In this process, psychological resilience could mitigate the adverse effects of body image disturbances on social avoidance by enhancing individuals’ adaptability. Burn patients with high family support often exhibited more positive cognitions of their body image, enhanced their psychological resilience, and improved their social avoidance.

**Conclusion:**

This study further deepened and expanded the fear-avoidance model by proposing a collaborative intervention framework involving family support and psychological resilience. These findings offer new practical suggestions for clinical practitioners and family members to enhance the interpersonal communication skills of burn patients.

## 1 Introduction

Burn injuries, as a severe type of trauma, have profound implications for both physical and psychological health (1, 2). Burns are the fourth most common type of injury globally, following only traffic accidents, falls, and interpersonal violence (3). Annually, approximately 9 million people worldwide die from burns, and millions more suffer permanent injuries (4). Burns not only cause permanent damage to the skin and tissues but also significantly impact patients’ body image (5). Body image, defined as an individual’s perception and evaluation of their physical appearance and functionality, often undergoes significant changes after burns (6, 7). According to the body image nursing theory, body image is dynamically constituted by three elements: actual physical condition, physical presentation, and ideal physical state, and imbalances among these can lead to body image disturbances (8). Research has shown that burn patients often develop distorted self-perceptions due to scarring, skin pigmentation, and functional losses, leading to body image disturbances (9–11). These disturbances often manifest as excessive preoccupation with appearance, depression, shame, and social anxiety (12–14). For instance, Huang and Su (15) found a significant positive correlation between the severity of burns and the degree of body image disturbances in patients. However, recent studies have shown that body image disturbances are not only related to burn severity but also influenced by coping strategies, social support networks, and the rehabilitation environment (16–18). For example, patients with burns on exposed body parts, such as the face or limbs, are more likely to develop persistent self-aversion due to changes in appearance, leading to avoidance of mirrors, social gatherings, and even daily activities (19). Clearly, this disturbance not only affects patients’ self-esteem but also poses long-term negative impacts on their social interactions and psychological health.

Social avoidance is a manifestation of deficits in social interaction skills (20). It refers to behaviors where individuals, driven by self-protective or emotional discomfort motives, actively avoid social situations or interpersonal interactions (21, 22). Previous studies have identified factors such as active behavioral avoidance, emotional anxiety, catastrophizing thoughts, and self-doubt as characteristic of this behavior pattern (23, 24). For instance, Yuan et al. (25) found that patients’ social avoidance further exacerbated symptoms of anxiety and depression. Among burn patients, tendencies toward social avoidance are particularly pronounced. Clinical observations indicate that approximately 40% of moderate to severe burn patients exhibit significant social withdrawal and depressive behavior within one year post-injury (26, 27), manifesting as reduced interpersonal contact, refusal to participate in public activities, and even complete withdrawal from social and family circles (28, 29). This avoidance is not merely a result of emotional distress but is closely associated with the negative self-perceptions stemming from body image disturbances. In other words, burn patients are more likely to adopt avoidance strategies, such as limiting social activities or concealing scars, to alleviate anxiety related to negative evaluations. While this avoidance temporarily reduces suffering, it perpetuates long-term isolation, hindering reintegration into social roles and creating a cyclical pattern of psychosocial decline. However, previous studies have primarily focused on the adverse effects of social avoidance or body image disturbances in isolation, with little attention paid to their relationship or to burn patients as a unique population. Thus, this study aims to address the following questions: (1) Does body image disturbance in burn patients have a negative impact on social avoidance? (2) What are the internal mechanisms and boundary conditions of the relationship between body image disturbance and social avoidance?

Psychological resilience, defined as a core protective factor for coping with adversity (30, 31), refers to an individual’s ability to effectively cope with, adapt to, and even thrive in the face of life stress, trauma, or significant life changes (32, 33). Initially conceptualized to describe resilience in children and adolescents in adverse conditions (34), its application has expanded to include all age groups, particularly in the medical field (35, 36). Research indicates that psychological resilience not only helps individuals mitigate the impact of negative emotions but also promotes the generation of positive emotions, thereby enhancing overall psychological and physical health (37). In the context of burns, Kornhaber et al. (38) found that burn patients with higher psychological resilience exhibited better coping abilities and more positive psychological states when facing challenges such as pain, delayed wound healing, and long-term care. Although this study highlighted the relationship between psychological resilience and psychological adaptation in burn patients, it did not further explore body image disturbances. Body image disturbances refer to negative cognitions and emotional reactions to one’s appearance and functionality (39, 40). Psychological resilience plays a crucial role in mitigating body image disturbances. On one hand, resilience enables individuals to reassess their physical changes more positively, reducing excessive preoccupation with appearance (41). For example, Tehranineshat et al. (42) found that burn patients with higher psychological resilience were more likely to focus on functional recovery and quality of life rather than purely cosmetic recovery. This suggests that resilience may help patients reconstruct a positive body image. On the other hand, psychological resilience can alleviate the negative effects of body image disturbances by enhancing self-efficacy and hope levels (43). Furthermore, body image disturbances in burn patients can reduce social avoidance by increasing psychological resilience. According to psychological resilience theory, individuals with higher resilience exhibit stronger emotional regulation and problem-solving skills, enabling them to reconstruct negative self-perceptions and actively engage in social challenges (44). Thus, psychological resilience can buffer the impact of body image disturbances by promoting acceptance of physical changes and reducing avoidance-driven behaviors.

Family support refers to the help and companionship provided by family members through emotional, informational, evaluative, and tangible means (45). In the field of burn rehabilitation, family support is critical in alleviating severe psychological and social adaptation problems. According to the stress-appraisal theory, family support can help patients better cope with the challenges of their condition and rehabilitation by relieving anxiety and depression (46). Recent studies further indicate that family support can influence patients’ psychological health (47, 48) and indirectly affect social behaviors by regulating cognitive appraisals and behavioral responses (49). This is primarily because family support provides emotional comfort and a sense of acceptance, helping patients redefine their body image in a positive light. For example, Ma and Zhang (50) found a significant positive correlation between high levels of family support and patients’ satisfaction with their body image. Additionally, family support can provide patients with the necessary information and resources to help them better cope with physical changes. For instance, Shokre et al. (3) pointed out that the positive attitudes and encouragement of family members can significantly improve burn patients’ body image and reduce anxiety about physical disfigurement.

Based on the fear-avoidance model, this study proposes and tests a regulatory mediation model to elucidate how family support moderates the mediating role of psychological resilience in the relationship between body image disturbances and social avoidance. The core theoretical pathway begins with individuals’ negative cognitions and emotional evaluations of their body image, which are often viewed as potentially harmful internal stimuli that may trigger the fear-avoidance cycle (6, 7, 51). According to the fear-avoidance model, if individuals misinterpret such discomfort as a significant threat that they are unable to cope with (e.g., believing their appearance will inevitably lead to ridicule or rejection), they are likely to experience heightened distress and fear (52, 53). This fear becomes a key driver, prompting individuals to adopt avoidance strategies, such as avoiding social gatherings or refusing activities that involve bodily exposure, in an effort to reduce short-term distress (54, 55). However, these avoidance behaviors are maladaptive in the long run (56), as they deprive individuals of opportunities to engage in positive social interactions, challenge negative beliefs, or develop effective coping strategies. Instead, they perpetuate or exacerbate body image disturbances and social anxiety, creating a vicious cycle (57). In this critical pathway, psychological resilience emerges as a key internal resource for breaking the vicious cycle, reflecting individuals’ ability to maintain functional stability, adapt flexibly, and recover from body image-related stress. Highly resilient individuals are more likely to adopt adaptive rather than avoidant coping strategies, such as cognitive restructuring, seeking support, or persisting in meaningful activities, thereby weakening the direct pathway from body image disturbances to social avoidance via fear.

The regulatory role of family support acts on two critical nodes in the mediating pathway of psychological resilience. First, during the stage in which body image disturbances erode psychological resilience resources, high-quality family support can effectively buffer the negative impact of body image disturbances on resilience. This is manifested through unconditional acceptance, emotional warmth, constructive communication, and affirmation of individual worth. When individuals perceive their family as a source of safety and affirmation, their feelings of shame and worthlessness resulting from body dissatisfaction are significantly reduced. This protects their psychological energy reserves, enabling them to mobilize internal resources rather than becoming depleted.

Second, during the stage in which psychological resilience is translated into actual coping behaviors and resistance to avoidance tendencies, family support plays an enhancing role. In environments with strong family support, even individuals with moderate initial levels of psychological resilience are more likely to receive immediate encouragement, emotional comfort, and problem-solving assistance when attempting to use adaptive coping strategies (58). This significantly enhances the reinforcing effects of adaptive coping, thereby increasing the effectiveness of psychological resilience in reducing avoidance behaviors (59). In contrast, in low-support or critical family environments, individuals, even if they possess a certain level of psychological resilience, may find their efforts to engage in social activities thwarted due to a lack of reinforcement, additional family pressures, or conflicts, increasing the risk of reverting to avoidance behaviors. However, this regulatory effect is particularly pronounced in individuals with high body image disturbances. Specifically, among those who experience high levels of perceived teasing, individuals with high perceived family support exhibit significantly higher levels of psychological resilience compared to those with low support (60). The cognitive behavioral model emphasizes that non-judgmental feedback and encouragement of positive bodily experiences from family are key external factors in reshaping body-related cognitions and reducing avoidance (61, 62). Thus, family support not only protects psychological resilience resources from being overly eroded by body image disturbances but also provides the necessary external catalysts and safety foundation for translating psychological resilience into positive, non-avoidant social coping behaviors. This strengthens the regulatory effects of family support in the entire body image disturbance-psychological resilience-social avoidance mediating chain, ultimately weakening the intensity of the fear-avoidance-maintenance vicious cycle driven by perceived threats to body image. This regulatory mediating mechanism provides essential targets for interventions aimed at addressing body image concerns and related social functioning impairments: enhancing family support systems, particularly by improving their responsiveness and effectiveness, can serve as a key strategy to enhance psychological resilience and disrupt the fear-avoidance cycle.

Based on the above analysis, this study proposes the following hypotheses:

H1: Body image disturbances in burn patients have a significant negative impact on social avoidance.
H2: Psychological resilience mediates the relationship between body image disturbances and social avoidance in burn patients.
H3: Family support moderates the relationship between body image disturbances and social avoidance.
H4: Family support moderates the mediating role of psychological resilience (left branch).

### 1.1 Research gap and novelty

Despite growing interest in body image and psychosocial functioning among burn survivors, three significant research gaps persist.

First, the field lacks an integrated theoretical framework explaining how body image disturbances translate into social avoidance. Existing studies tend to examine isolated associations, such as the effect of body image on emotional well-being or the consequences of social withdrawal (17, 63). Yet, no study has systematically interpreted the emergence of social avoidance from the perspective of internalized appearance-related threat (64). Drawing on the fear-avoidance model, the present study conceptualizes body image disturbance as an internally generated social-evaluative threat that may activate fear and avoidance cycles. This extends the model beyond its traditional pain-related domain and introduces a novel application in populations with appearance-related trauma.

Second, the mediating role of psychological resilience in this pathway has not been empirically tested among burn patients. Although resilience has been recognized as essential for recovery from trauma and chronic stress (65), it remains unclear whether it can buffer the impact of negative body image evaluations and prevent the escalation toward avoidance behavior. This study addresses this gap by identifying psychological resilience as a key mechanism linking body image disturbance to social avoidance.

Third, previous studies have largely overlooked the potential moderating role of family support in shaping these psychological processes. While family support has been shown to improve emotional outcomes in burn survivors directly (66), its role as a contextual moderator — specifically, whether it protects resilience from being eroded by body image disturbance and whether it enhances the adaptive impact of resilience on avoidance — remains unexplored. The present study employs a moderated mediation model to elucidate how family support operates at two critical stages, providing a more ecologically valid understanding of the recovery process.

By addressing these theoretical and empirical gaps, the present study proposes and tests a moderated mediation model that advances understanding of social avoidance in burn survivors and offers novel guidance for clinical interventions.

## 2 Method

### 2.1 Methodological rationale for study design

The present study employed a cross-sectional design, an approach widely used in rehabilitation and clinical psychology to examine theoretical associations and psychological mechanisms within complex, multi-variable systems (67, 68). The decision to employ a cross-sectional framework was primarily guided by the exploratory objective of this research—to empirically test a mediated and moderated model grounded in the fear-avoidance framework among burn patients. This population remains underrepresented in empirical psychology. Because the fear-avoidance model involves several latent psychosocial constructs, an initial cross-sectional design provides an efficient and theoretically valid means of identifying the strength and direction of associations among these constructs before conducting more resource-intensive longitudinal or experimental investigations.

Methodologically, the use of random sampling across two major burn rehabilitation centers, strict inclusion and exclusion criteria, and validated psychometric instruments substantially enhanced the internal validity and measurement reliability of our findings. Moreover, several procedural controls—such as the anonymized data collection process, Harman’s single-factor test for common method bias, and bootstrapping in the PROCESS macro for mediation and moderation analysis—served to strengthen the robustness of statistical inference despite the cross-sectional nature of the data.

We acknowledge that the cross-sectional design precludes definitive causal inference. However, given the exploratory stage of this research and the absence of existing integrated models addressing body image disturbance, resilience, and family support in burn populations, a cross-sectional approach represents a theoretically justified and pragmatic first step. Future research will employ prospective longitudinal and intervention-based designs to validate the causal pathways suggested by the present results and to explore temporal dynamics in the adaptation process following burn trauma.

### 2.2 Sample source

To validate the four hypotheses outlined above, this study conducted a cross-sectional investigation. Data collection was carried out between April 2025 and June 2025 at the Affiliated Hospital of North Sichuan Medical College and Nanchong Central Hospital. These two hospitals collectively serve as major referral institutions for burn and reconstructive surgery in southwestern China, providing coverage for both urban and rural catchment areas with diverse socioeconomic and demographic backgrounds. The Affiliated Hospital of North Sichuan Medical College represents a teaching hospital that primarily admits patients from northern and central Sichuan cities. In contrast, Nanchong Central Hospital serves as a regional comprehensive medical center, catering to extensive rural populations from the surrounding counties. The combination of these two hospitals, therefore, ensures representation across different socioeconomic strata, geographical origins, and clinical severities of burn injuries.

A multi-stage random sampling strategy was employed. First, within each hospital, the burn and plastic surgery departments were approached after obtaining ethical approval. Second, patient lists were generated during the data collection period, and eligible participants were randomly selected using a computer-generated randomization sequence stratified by gender and burn severity to avoid sampling bias. Third, both inpatient and outpatient cases were included to capture variability in treatment stage and rehabilitation context.

Upon obtaining their consent, we used random sampling to recruit participants. Subsequently, we informed the patients of the study’s purpose, potential risks, benefits, and data collection process. After obtaining their consent, they signed an informed consent form. Patients who owned mobile electronic devices and were capable of using them completed electronic questionnaires, while those without access to such devices or the ability to use them filled out paper-based questionnaires. Finally, all questionnaires (both electronic and paper-based) were collected and organized by the researchers. This study was reviewed and approved by the Medical Ethics Committee of Nanchong Central Hospital (Approval number: 2025116).

The minimum sample size required for this study was calculated using G*Power 3.1 software. We selected the F-test under multiple linear regression, specifically for the fixed model and R² change analysis. The significance level (α) was set to 0.05, the desired power (1-β) to 0.8, and the effect size (f²) to 0.15. The results indicated a minimum sample size of 119 participants. A total of 300 participants were recruited for this study, satisfying the minimum sample size requirement.

During the recruitment process, we established the following exclusion criteria for patients: (1) Refusal to sign the informed consent form. (2) Severe verbal expression impairments or cognitive functional disabilities. (3) Participation in similar investigations within the past month. (4) Compliance with the Burn Clinical Diagnostic and Treatment Guidelines, and diagnosed by a burn specialist. (5) Total burn area ≥10% for adults or ≥5% for children. (6) History of severe mental illness. (7) Questionnaire completion time exceeding 8 minutes and exclusion of strongly consistent responses. For the assessment of cognitive functional impairments, this study utilized the Mini-Mental State Examination (MMSE), with a total score of 23 or below as the criterion (69).

In addition to self−report measures, several objective clinical variables were collected by attending burn specialists to characterize burn severity and scarring outcomes. These included the burn cause (thermal, flame, chemical, electrical), burn stage (infection, acute exudative, repair), and whether surgical reconstruction or grafting was performed. The classification of burn severity (mild, moderate, severe, extremely severe) followed the Chinese Burn Society Clinical Diagnostic and Treatment Guidelines (2024 edition) (70). Collecting these indicators enabled us to examine differences in body-image disturbance across clinical categories statistically and to control for the possible influence of burn severity on self-perceived body image. Although indices such as the Vancouver Scar Scale (VSS) (71) or Patient and Observer Scar Assessment Scale (POSAS) (72) were not incorporated due to patient-privacy concerns and variability in wound-healing time, the clinical variables above provide objective anchors complementing subjective perceptions. Future research will integrate standardized scar visibility assessments, such as trained clinician ratings or three-dimensional imaging, to triangulate the objective and perceived dimensions of body image disturbance.

**Figure 1:**
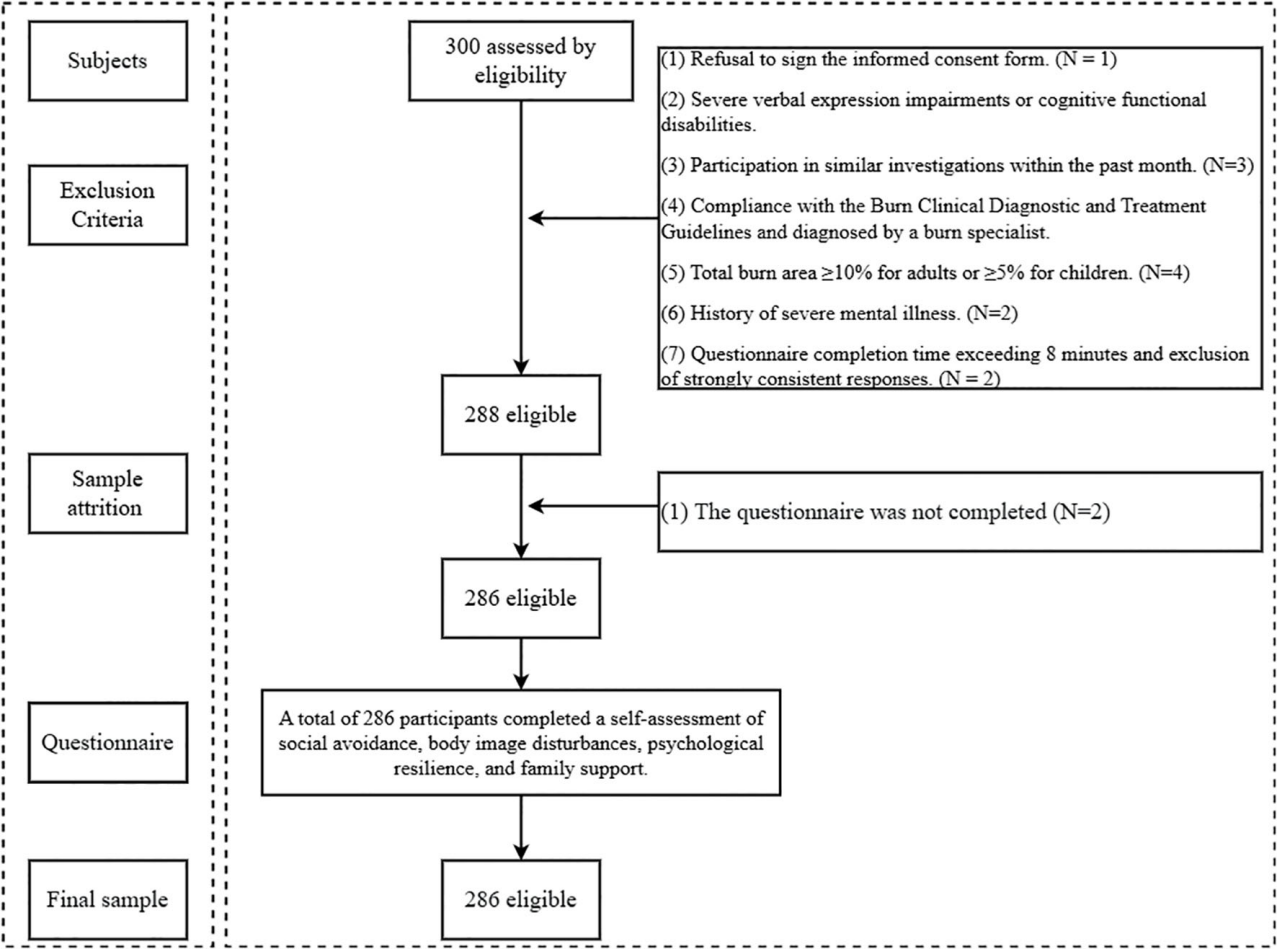
Data screening procedure.

During the data collection and cleaning stages, we excluded 14 participants based on the aforementioned criteria (see **Figure 1** for details). The final effective sample size was 286, with an effective rate of 95.33%. Of these, 173 were male burn patients and 113 were female burn patients. The age distribution of the burn patients was approximately 28.99 ± 8.78 years. Further demographic details are provided in **Table 1**.

**Table 1:** Demographic information of burn patients.

Variables	Items	Number	Proportion
Gender	Male	173	60.5%
	Female	113	39.5%
Education Background	Primary School	9	3.1%
	Junior High School	51	17.8%
	Senior High School/College	89	31.1%
	Undergraduate	120	42%
	Master’s Degree	13	4.5%
	Doctoral Degree	4	1.4%
Marital Status	Unmarried	113	39.5%
	Married	139	48.6%
	Divorced	30	10.5%
	Widowed	4	1.4%
Occupation	Students	36	12.6%
	Employee	132	46.2%
	Unemployed	36	12.6%
	Worker	35	12.2%
	Freelancer	42	14.7%
	Retired	5	1.7%
Children	Yes.	143	50%
	No.	143	50%
Residence	Urban	196	68.5%
	Rural	90	31.5%
Income level	≤ 1,000 yuan	23	8.0%
	1,001-3,000 yuan	61	21.3%
	3,001-5,000 yuan	110	38.5%
	5,001-8,000 yuan	71	24.8%
	Above 8,000 yuan	21	7.3%
Cause of Burn Heat	Overheated materials	88	30.8%
	Flame	127	44.4%
	Electrical Burn	61	21.3%
	Chemical Burn	10	3.5%
Burn Severity	Mild Burn	141	49.3%
	Moderate Burn	82	28.7%
	Severe Burn	54	18.9%
	Extremely Severe Burn	9	3.1%
Burn Stage	Infection Stage	97	33.9%
	Acute Exudative Stage	85	29.7%
	Repair Stage	104	36.4%
Burn Surgery	Yes.	132	46.2%
	No.	154	53.8%

### 2.3 Measurement tools

Prior to formal data collection, all measures underwent a standardized cross-cultural adaptation process to ensure conceptual and linguistic equivalence for the Chinese burn-patient population. Each instrument originally developed in English was first translated and then back-translated by two independent bilingual psychologists, following the guidelines of Wen et al. (73) and Brislin (74). A panel of three experts in clinical psychology and burn rehabilitation then reviewed the preliminary Chinese versions to confirm semantic, idiomatic, and conceptual equivalence. A pilot test was subsequently conducted with 32 burn patients (who were not included in the final sample) to assess the clarity and comprehension of the items. Based on feedback, minor linguistic revisions were made to improve readability.

All adapted scales demonstrated excellent psychometric properties within the current sample. Confirmatory factor analyses (CFAs) were performed for each scale, yielding acceptable to excellent model fits (CFI > 0.90, TLI > 0.90, RMSEA < 0.08). Reliability coefficients were strong for all constructs—Body Image Disturbance (α = 0.930), Psychological Resilience (α = 0.939), Family Support (α = 0.870), and Social Avoidance (α = 0.929). These results confirm that the adapted measures are culturally valid and psychometrically robust for assessing Chinese burn survivors.

#### 2.3.1 Social avoidance scale

The Social Avoidance Scale, adapted from Ottenbreit and Dobson (75), consists of 31 items across four dimensions: behavioral social avoidance, behavioral nonsocial avoidance, cognitive nonsocial avoidance, and cognitive social avoidance. For this study, we selected the behavioral social avoidance and cognitive social avoidance dimensions to measure social avoidance in burn patients, comprising 15 items (e.g., “I avoid participating in social activities”). A Likert 7-point scale was used for scoring (1=“Strongly Disagree,” 7=“Strongly Agree”), with higher scores indicating stronger tendencies toward social avoidance. The Cronbach’s alpha coefficient for the social avoidance scale in this study was 0.929, indicating good reliability.

#### 2.3.2 Body image disturbance scale

The Body Image Disturbance Scale was adapted from Lawrence et al. (76) and consists of 14 items. This study used all 14 items to assess body image disturbances in burn patients (e.g., “Due to burn-related appearance changes, I feel uncomfortable in front of my family”). The scale has been widely used in Chinese burn patient populations and demonstrates excellent cultural adaptability, as seen in Huang and Su (15), who used this scale to assess body image disturbances in 115 burn patients. A Likert 7-point scale was used for scoring (1=“Strongly Disagree,” 7=“Strongly Agree”), with higher scores indicating greater body image disturbances. Following model fit and reliability testing, the Body Image Disturbance Scale used in this study demonstrated good psychometric properties (Cronbach’s α=0.930).

#### 2.3.3 Family support scale

The Family Support Scale for burn patients was adapted from Zimet et al. (77) Multidimensional Scale of Perceived Social Support, comprising 12 items across three dimensions: significant others, family, and friends. This study selected the family dimension to assess family support, consisting of 4 items (e.g., “I receive emotional help and support from my family”). The scale was translated into Chinese by Yang et al. (78) and validated for psychometric properties and cultural adaptability. The Cronbach’s alpha coefficient for the family support scale in this study was 0.870, indicating good reliability. A Likert 7-point scale was used for scoring (1=“Strongly Disagree,” 7=“Strongly Agree”), with higher scores indicating greater perceived family support.

#### 2.3.4 Psychological resilience scale

The Psychological Resilience Scale for burn patients was adapted from Lock et al. (79) resilience scale, which assesses both state and trait dimensions. The scale consists of 13 items, and this study used all 13 items to assess psychological resilience in burn patients. The scale has been widely used by Chinese scholars to evaluate resilience in Chinese populations (80, 81), demonstrating good cultural adaptability and psychometric properties. A Likert 7-point scale was used for scoring (1=“Strongly Disagree,” 7=“Strongly Agree”), with higher scores indicating greater psychological resilience. The psychological resilience scale used in this study demonstrated good psychometric properties (Cronbach’s α=0.939).

### 2.4 Data analysis

Firstly, this study used skewness and kurtosis to assess whether the data sample conformed to an approximately normal distribution. Next, Harman’s single-factor test was performed to examine whether the model exhibited severe common method bias. Subsequently, correlation analysis and descriptive statistics were used to analyze the linear relationships between the variables. Fourth, the mediating role of psychological resilience was analyzed using the SPSS (V 27.0) macro program’s Process Model 4. Fifth, the moderating effect of family support was analyzed using Process Model 1. Sixth, the moderating effect of family support on the mediating role of psychological resilience was analyzed using Process Model 8.

## 3 Results

### 3.1 One-way ANOVA of demographic information and social avoidance

We used demographic information as independent variables and social avoidance as the dependent variable, and conducted a one-way ANOVA, as shown in **Table 2**. The results indicated significant differences in social avoidance among burn stages (F=5.016, p=0.007, η²=0.034) and burn surgeries (F=21.356, p < 0.001, η²=0.070). However, there were no significant differences in social avoidance among the genders, educational backgrounds, marital statuses, and other demographic information (p > 0.05).

**Table 2:** One-way ANOVA of Demographic Information and Social Avoidance.

Variables	Items	M	SD	F	p	η2
Gender	Male	4.151	1.326	0.093	0.761	0.001
	Female	4.109	1.167			
Education Background	Primary School	3.304	0.893	1.328	0.252	0.023
	Junior High School	4.112	0.992			
	Senior High School/College	4.227	1.114			
	Undergraduate	4.175	1.233			
	Master’s Degree	3.949	1.241			
	Doctoral Degree	3.633	0.451			
Marital Status	Unmarried	4.284	1.171	1.775	0.152	0.019
	Married	4.091	1.120			
	Divorced	3.871	1.148			
	Widowed	3.417	0.725			
Occupation	Students	3.850	1.332	1.582	0.165	0.027
	Employee	4.252	1.092			
	Unemployed	3.852	0.883			
	Worker	4.390	1.000			
	Freelancer	4.041	1.384			
	Retired	4.133	1.124			
Children	Yes.	4.108	1.118	0.159	0.69	0.001
	No.	4.162	1.174			
Residence	Urban	4.151	1.148	0.131	0.718	0.001
	Rural	4.099	1.142			
Income level	≤ 1,000 yuan	3.687	1.174	1.257	0.287	0.018
	1,001-3,000 yuan	4.042	1.178			
	3,001-5,000 yuan	4.196	1.005			
	5,001-8,000 yuan	4.220	1.132			
	Above 8,000 yuan	4.289	1.635			
Cause of Burn Heat	Overheated materials	3.992	1.280	0.74	0.529	0.008
	Flame	4.171	1.135			
	Electrical Burn	4.260	0.962			
	Chemical Burn	4.160	1.055			
Burn Severity	Mild Burn	4.084	1.261	0.843	0.471	0.009
	Moderate Burn	4.121	1.048			
	Severe Burn	4.336	1.029			
	Extremely Severe Burn	3.844	0.549			
Burn Stage	Infection Stage	4.227	1.045	5.016	0.007	0.034
	Acute Exudative Stage	4.361	1.215			
	Repair Stage	3.864	1.132			
Burn Surgery	Yes.	4.461	1.125	21.356	<0.001	0.070
	No.	3.854	1.088			

### 3.2 Common method bias analysis

All questionnaires were based on self-reported data, and Harman’s single-factor test was used to examine potential common method bias. The results revealed 6 factors with eigenvalues greater than 1, with the first factor explaining 32.286% of the variance, which did not exceed the critical threshold of 40%. This indicates that no severe common method bias was present in this study. Furthermore, anonymous data collection methods were employed during the survey process to further reduce the likelihood of common method bias.

### 3.3 Correlation analysis

The normality test included tests for kurtosis and skewness, primarily to assess the symmetry and peakedness of the data. Kline (82) suggests that skewness values between ±3 and kurtosis values between ±8 indicate that the sample data are consistent with a normal distribution. According to the results presented in **Table 3**, the data in this study were normally distributed.

**Table 3:** Normality test of variables.

Variables	M	SD	Skewness	Kurtosis	1	2	3	4
1. Body Image Disturbance	3.889	1.247	-0.004	-0.418	1			
2. Psychological Resilience	4.263	1.323	-0.381	0.592	-0.339**	1		
3. Family Support	4.627	1.489	-0.455	-0.043	-0.142*	0.240**	1	
4. Social Avoidance	4.135	1.145	-0.036	-0.152	0.646**	-0.391**	-0.208**	1

**p < 0.01; *p < 0.05.

Correlation analysis was conducted on body image disturbances, social avoidance, family support, and psychological resilience, as shown in **Table 3**. The results indicated the following significant correlations: Body image disturbances were significantly negatively correlated with psychological resilience (r=-0.339, P < 0.01). Body image disturbances were significantly negatively correlated with family support (r=-0.142, P < 0.05). Body image disturbances were significantly positively correlated with social avoidance (r=0.646, P < 0.01). Psychological resilience was significantly positively correlated with family support (r=0.240, P < 0.01). Psychological resilience was significantly negatively correlated with social avoidance (r=-0.391, P < 0.01). Family support was significantly negatively correlated with social avoidance (r=-0.208, P < 0.01).

### 3.4 Mediation effect of psychological resilience

To further examine the mediating role of psychological resilience in the relationship between body image disturbances and social avoidance, and to test Hypothesis H2, this study treated psychological resilience as the mediator, body image disturbances as the independent variable, and social avoidance as the dependent variable. The mediating effect was analyzed using Process Model 4 (Bootstrap sample: 5000) (83). The results showed that: Body image disturbances had a significant impact on psychological resilience (β=-0.359, 95% CI=[-0.476, -0.243], P < 0.001). Body image disturbances had a significant impact on social avoidance (β=0.532, 95% CI=[0.448,0.617], P < 0.001). Psychological resilience had a significant impact on social avoidance (β=-0.168, 95% CI=[0.448,0.617], P < 0.001). Overall, psychological resilience partially mediated the relationship between body image disturbances and social avoidance (β=0.061, SE=0.022, 95% CI=[0.025, 0.1099]), as shown in **Table 4**, supporting Hypothesis H2.

**Table 4:** Table of regression coefficients for the mediating effect of resilience.

Regression equation		Overall fit index			Significance of regression coefficient		
Outcome variables	Predictive variables	R	R2	F	β	t	95% CI
Psychological resilience	Body image disturbances	0.339	0.115	36.971***	-0.359	-6.081***	[-0.476,-0.243]
Social avoidance	Body image disturbances	0.672	0.451	116.373***	0.532	12.403***	[0.448,0.617]
	Psychological resilience				-0.168	-4.146***	[-0.248,-0.088]

***p < 0.001.

### 3.5 Moderation effect of family support

**Figure 2:**
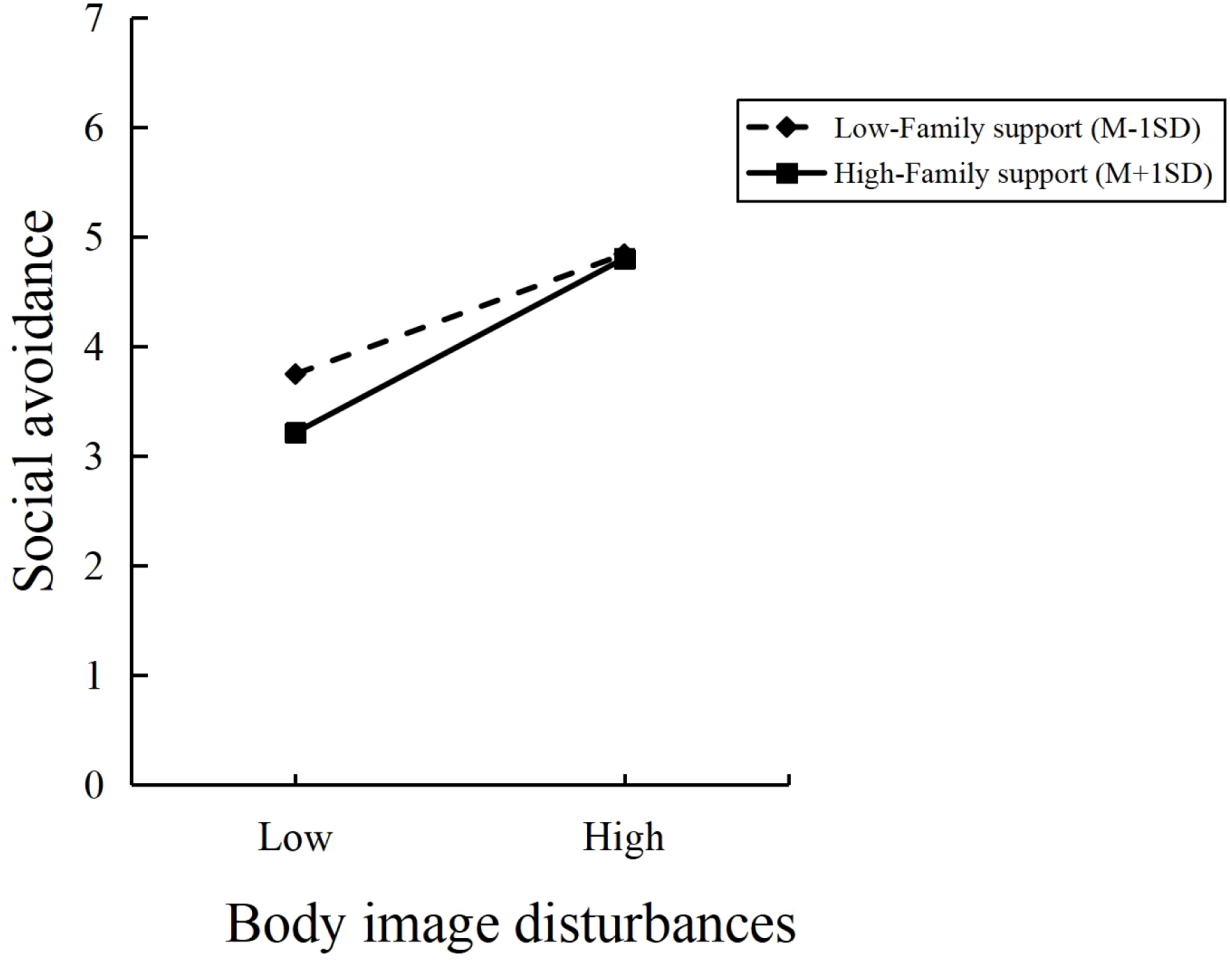
Results plot of the interaction effects of body image disturbances and family support.

Next, family support was treated as the moderator, body image disturbances as the independent variable, and social avoidance as the dependent variable. The moderating effect of family support was analyzed using Process Model 1 (Bootstrap sample: 5000) (83). The results indicated that: Family support had a significant impact on social avoidance (β=-0.096, 95% CI=[-0.164, -0.029], p=0.005). Body image disturbances had a significant impact on social avoidance (β=0.538, 95% CI=[0.452, 0.623], p < 0.001). The interaction between family support and body image disturbances had a significant impact on social avoidance (β=0.067, 95% CI=[0.019, 0.113], p < 0.001). Thus, family support significantly moderated the relationship between body image disturbances and social avoidance, as shown in **Figure 2**, supporting Hypothesis H3.

### 3.6 Moderated mediation effect

**Figure 3:**
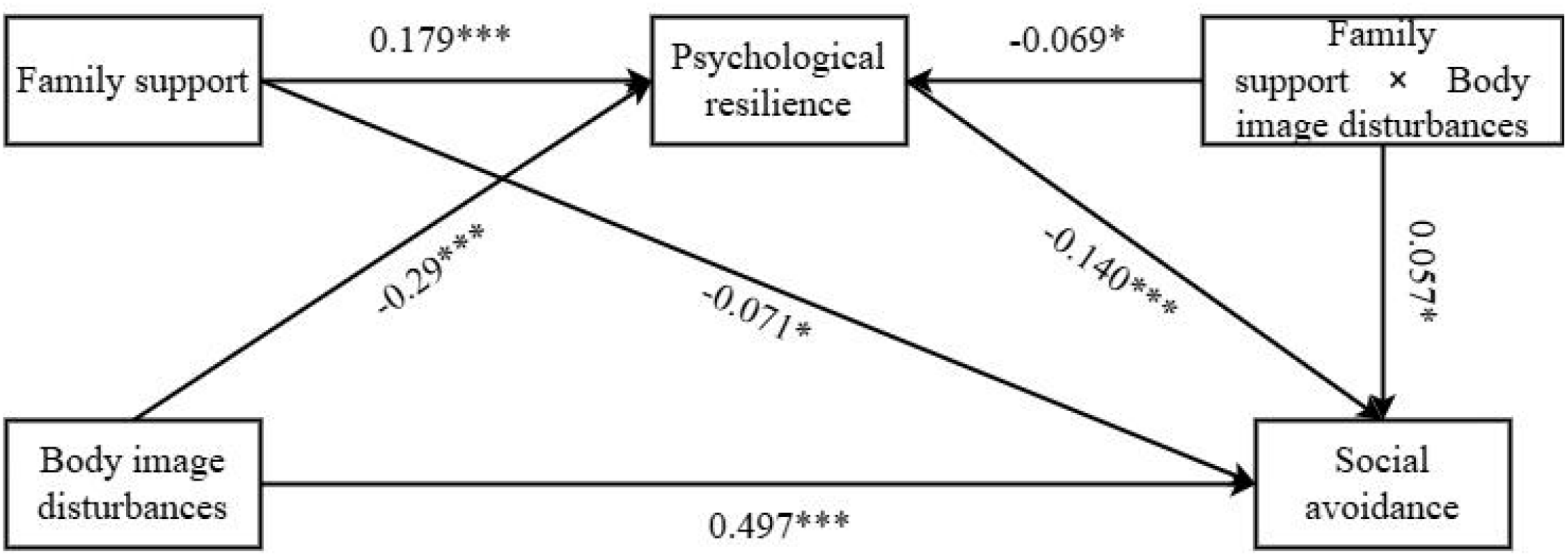
Plot of path coefficients for the moderated mediation model. *p < 0.05, ***p < 0.001.

Finally, the moderating effect of family support on the mediating role of psychological resilience was analyzed. Family support was treated as the moderator, psychological resilience as the mediator, body image disturbances as the independent variable, and social avoidance as the dependent variable. The moderated mediation effect was examined using Process Model 8 (Bootstrap sample: 5000) (83). The results showed that Body image disturbances had a significant impact on psychological resilience (β=-0.290, 95% CI=[-0.411, -0.169], p < 0.001). Body image disturbances had a significant impact on social avoidance (β=0.497, 95% CI=[0.409,0.584], p < 0.001). Psychological resilience had a significant impact on social avoidance (β=-0.140, 95% CI=[-0.221, -0.059], p < 0.001). Family support had a significant impact on social avoidance (β=-0.071, 95% CI=[-0.139, -0.003], p=0.04). Family support had a significant impact on psychological resilience (β=0.179, 95% CI=[0.082, 0.275], p < 0.001). The interaction between family support and body image disturbances had a significant impact on social avoidance (β=0.057, 95% CI=[0.011,0.103], p < 0.05). The interaction between family support and body image disturbances had a significant impact on psychological resilience (β=-0.069, 95% CI=[-0.133, -0.004], p < 0.05). Overall, family support effectively moderated the mediating role of psychological resilience in the relationship between body image disturbances and social avoidance. As shown in **Figure 3** and **Table 5**, this supports Hypothesis H4.

**Table 5:** Table of regression coefficients path for the moderated mediation model.

Regression equation		Overall fit index			Significance of regression coefficient		
Outcome variables	Predictive variables	*R*	*R2*	*F*	*β*	*t*	*95%CI*
Psychological resilience	Body image disturbances	0.405	0.164	18.464***	-0.290	-4.704***	[-0.411, -0.169]
	Family support				0.179	3.649***	[0.082,0.275]
	Family support × Body image disturbances				-0.069	-1.981*	[-0.133, -0.004]
Social avoidance	Body image disturbances	0.685	0.469	62.089***	0.497	11.225***	[0.409,0.584]
	Psychological resilience				-0.140	-3.413***	[-0.221, -0.059]
	Family support				-0.071	-2.063*	[-0.139, -0.003]
	Family support × Body image disturbances				0.057	2.432*	[0.011,0.103]

*p < 0.05, ***p < 0.001.

## 4 Discussion

The present study investigated how body image disturbances contribute to social avoidance among burn survivors and demonstrated a moderated mediation mechanism in which psychological resilience serves as a key mediator and family support functions as a contextual buffer. These findings not only substantiate prior theoretical work on body image and trauma adaptation but also extend the applicability of the fear−avoidance framework to appearance-related conditions. Furthermore, they provide important clinical implications for rehabilitation programs in burn care.

### 4.1 Theoretical significance

#### 4.1.1 The negative impact of body image disturbances on social avoidance

This study’s finding that body image disturbances in burn patients have a significant negative impact on social avoidance holds profound theoretical implications. First, this discovery substantially extends research in the field of social psychology concerning the relationship between body image and social behavior. Traditional studies have primarily focused on the negative effects of body image disturbances on psychological health, such as reduced self-worth and increased depressive symptoms (18, 84), while paying less attention to the specific mechanisms by which body image disturbances influence social behavior. However, through the analysis of cross-sectional data, this study reveals that body image disturbances not only affect psychological states but also profoundly impact social behavior, particularly by influencing social avoidance and thereby affecting an individual’s level of social integration. This shift in research perspective provides a new theoretical framework for social psychology and lays a theoretical foundation for a deeper understanding of social adaptation issues in burn patients. Within the framework of social learning theory, this study provides evidence to validate the core hypothesis that self-perceptions guide behavior. Body image disturbances, as a core dimension of self-perception, influence individuals’ negative self-evaluations, which in turn lead to a tendency to avoid social interactions. This not only enriches the application of social learning theory but also provides new theoretical entry points for related research.

The present study extends the classical fear-avoidance model by redefining the nature of the “threat” and the ensuing avoidance cycle in the context of burn trauma (85). While the original model conceptualizes threat as an externally derived pain-related stimulus (86), our findings highlight that burn patients often experience an internally generated cognitive-affective threat—namely, negative evaluation of their altered body image. This internalization transforms body image disturbances into a potent social-evaluative fear signal, activating avoidance not toward physical pain but toward perceived stigmatization and social exposure (87). By emphasizing appearance-based and interpersonal fear, this study refines trauma psychology’s understanding of avoidance from a pain-centric to a self-representation–centric model. This conceptual shift integrates body image research into the broader fear-avoidance framework, illustrating that maladaptive avoidance may arise from self-perceived bodily threats as much as from physical nociception.

#### 4.1.2 The mediating role of psychological resilience

Psychological resilience, an individual’s ability to exhibit positive adaptive capacity in the face of adversity, has recently become a focal point of research in psychology (88). However, studies on the specific mechanisms of psychological resilience in particular clinical populations, especially their mediating role in the relationship between body image disturbances and social behavior, remain in their early stages. This study is the first to explicitly identify the mediating role of psychological resilience in the relationship between body image disturbances and social avoidance in burn patients. Specifically, this finding demonstrates that psychological resilience can mitigate the negative effects of body image disturbances on social avoidance by enhancing individuals’ adaptive capabilities. This discovery not only enriches the application of resilience theory but also provides theoretical support for the development of resilience-based intervention strategies. The stress-adaptation model emphasizes the adaptive processes and outcomes individuals experience when facing stress, with psychological resilience being a core regulatory factor in this process. By revealing the mediating role of psychological resilience in specific stressful contexts, this study further validates the stress-adaptation model.

Prior literature has highlighted resilience as a buffer against trauma-related symptoms and chronic stress (89, 90), yet few studies have examined its role in pathways related specifically to appearance concerns. Our findings show that individuals with greater resilience are less likely to interpret their altered appearance catastrophically and more capable of reappraising distressing emotions, thus reducing avoidance tendencies. These results align with resilience frameworks, suggesting that adaptive coping and positive reappraisal can interrupt maladaptive cycles in the aftermath of trauma (44). Importantly, the mediating role identified here supports the conceptualization of resilience as a dynamic regulatory mechanism, rather than merely a personality trait, highlighting its potential as a target for clinical intervention in burn survivors.

Within this reconceptualized fear-avoidance model, psychological resilience serves as a core intrapersonal regulatory mechanism that dynamically mediates the pathway from body image disturbance to social avoidance. Unlike traditional resilience theories that treat resilience as a static trait (91), our moderated mediation structure portrays it as a state-like resource responsive to contextual modulation. This dynamic perspective refines trauma adaptation theory by demonstrating that resilience functions not merely as an outcome variable following adversity, but as an active disruptor in the fear–avoidance feedback loop. Specifically, higher resilience buffers the translation of body image–related fear into avoidance behavior, enabling cognitive reappraisal and social re-engagement. Thus, our model advances the theory of resilience by illustrating how adaptive regulation interacts with perceptual cognition to recalibrate behavioral responses in response to trauma-related self-threat.

#### 4.1.3 The moderating role of family support

This study revealed the moderating role of family support in the relationship between body image disturbances and social avoidance. Specifically, the findings showed that family support can mitigate the negative effects of body image disturbances on social avoidance by enhancing psychological resilience. This discovery provides theoretical support for the application of family support in the rehabilitation of burn patients. Traditional rehabilitation interventions have often focused on individual psychological or medical treatments, neglecting the importance of interpersonal interactions within the family in patient recovery (92). This study demonstrates that family support not only directly moderates the impact of body image disturbances on social avoidance but also indirectly influences this relationship by enhancing psychological resilience. The discovery of this dual regulatory mechanism further reveals the central role of family support in the psychological rehabilitation of burn patients. Thus, family support serves not only as an external resource for patient recovery but also as a means of strengthening internal psychological resources, thereby promoting psychological adaptation and social reintegration.

The moderating effects of family support at both stages of the mediation process underscore its multifaceted contribution to recovery. This finding is consistent with previous studies, which have shown that strong family involvement improves psychological well-being and social adjustment in patients with chronic conditions (93, 94). Our findings further refine this understanding by demonstrating that family support not only enhances the protective effect of resilience but also buffers against the depletion of psychological resources caused by body image disturbances. Family members who provide validation, emotional encouragement, and nonjudgmental communication may reduce patients’ feelings of shame and reinforce their motivation to engage in social activities (95). These results align with social-cognitive theories, which highlight the role of interpersonal feedback in shaping self-efficacy and behavioral responses (96). Thus, family support can serve as a key ecological factor that strengthens adaptive functioning and reduces avoidance behaviors among burn survivors.

Beyond the individual level, the inclusion of family support introduces an interpersonal regulatory layer that operates across the entire mediating chain. This contextual variable redefines the boundaries of the fear-avoidance model in two critical ways. First, it positions adaptation to trauma as ecologically embedded—situated within relational systems that provide feedback, reinforcement, and meaning reconstruction. Traditional trauma frameworks often neglect these relational moderators, focusing predominantly on individual coping. Our model addresses this limitation by empirically demonstrating that resilient adaptation is context-contingent, with familial responsiveness amplifying or constraining it. Second, by statistically modeling family support as a moderator of both the erosion of resilience and its translation into adaptive behavior, the present study synthesizes individual and systemic resilience perspectives. This dual-layer regulation elucidates how family support strengthens the buffering capacity of resilience, effectively interrupting the fear–avoidance–maintenance cycle that perpetuates social withdrawal in burn patients. Collectively, this refinement situates our framework as a bridge between body image literature and trauma recovery theory, offering a more comprehensive, contextually sensitive model of psychosocial adaptation.

#### 4.1.4 Comparative theoretical perspectives

To further situate the proposed model within broader theoretical traditions, it is useful to compare its explanatory logic with existing frameworks in trauma-related and body-image research, particularly the cognitive-behavioral and social-cognitive models.

Traditional cognitive-behavioral models of social avoidance emphasize the role of maladaptive cognitions and catastrophic beliefs about the self and others as direct antecedents of avoidance behavior (97). Within this framework, interventions focus on identifying and restructuring distorted beliefs to modify behavior. While our study is consistent with this premise, it refines the model by introducing psychological resilience as a dynamic adaptive filter between negative body image cognitions and avoidance outcomes. Rather than solely targeting cognitive distortion, the present model conceptualizes resilience as an emotion regulatory bridge that enables cognitive reappraisal and adaptive behavioral engagement. Moreover, the inclusion of family support acknowledges that cognitive restructuring does not occur in isolation but rather depends on a reinforcing environment that validates new self-perceptions. Thus, our findings extend the cognitive-behavioral perspective from a purely individual cognition-behavior linkage to a multi-systemic self-regulation framework that integrates personal and social dimensions.

According to social-cognitive theory (98), behavior arises from reciprocal interactions among personal factors, environmental contexts, and behavioral feedback processes. Central to this paradigm is self-efficacy, the belief in one’s ability to perform and succeed in social interactions. Our results converge with this model by demonstrating that psychological resilience enhances perceived efficacy in managing body-image threats, thereby reducing avoidance. Importantly, family support aligns with the environmental determinants in Bandura’s triadic reciprocity—functioning as a supportive context that fosters successful mastery experiences and vicarious encouragement. In this way, the moderated mediation model empirically operationalizes social-cognitive mechanisms through the joint action of internal resources (resilience) and external reinforcement (family support).

By synthesizing insights from these models, our study positions the fear-avoidance framework as a conceptual hub that links cognitive, affective, and contextual dimensions of trauma adaptation. While cognitive-behavioral theories emphasize thought correction, and social-cognitive models emphasize self-efficacy reconstruction, our moderated mediation model highlights the interactive regulation between cognitions and contexts through resilience and support systems. This conceptual integration advances trauma psychology beyond its traditional fear-response paradigm toward a multilevel adaptive systems perspective, in which internal capacity and external environment co-evolve to determine social reintegration after bodily trauma.

### 4.2 Clinical implications

The conclusions of this study hold significant implications for clinical practice, particularly in the rehabilitation and psychological support of burn patients. The finding that body image disturbances in burn patients have a significant positive influence on social avoidance provides important guidance for clinical practice. First, clinicians should not only focus on the physical recovery of burn patients but also pay special attention to changes in their body image. Early identification and assessment of body image disturbances can enable timely interventions to reduce social avoidance behaviors caused by these disturbances, thereby improving patients’ social adaptation abilities and quality of life. This discovery offers new directions for clinical interventions. For example, body image improvement programs can help patients rebuild positive body images, reducing the likelihood of social avoidance. Clinical psychologists and rehabilitation specialists can design targeted psychological interventions to help patients adjust their negative cognitions about their appearance and functionality, promoting their sense of social belonging and participation. These interventions not only improve patients’ psychological health but also lay the groundwork for their reintegration into society and social roles.

In burn patients, psychological resilience not only buffers the negative effects of body image disturbances on social avoidance but also promotes the rehabilitation process. Therefore, clinical practice should emphasize the cultivation and enhancement of psychological resilience. For instance, clinical psychologists can utilize psychological counseling and resilience training to help patients develop effective coping strategies and improve their ability to adapt to challenges. Specifically, resilience training programs, including mindfulness training, emotion regulation training, and the development of social support networks, can help patients exhibit stronger adaptive and recovery capabilities when facing body image disturbances. Additionally, clinical practitioners can use psychoeducation to help patients and their families understand the importance of psychological resilience and provide corresponding support and resources to address the challenges posed by body image disturbances. By enhancing patients’ psychological resilience, clinicians can reduce the likelihood of social avoidance and promote comprehensive rehabilitation and social integration.

The family, as the patient’s primary support system, plays a critical role in shaping their psychological state and behavioral outcomes. This study demonstrated that family support significantly moderates the impact of body image disturbances on social avoidance, highlighting its importance in patient rehabilitation. In clinical interventions, clinicians should pay special attention to the patient’s family support system and actively intervene to enhance support levels. On the one hand, clinicians can utilize family psychotherapy to help family members better understand the patient’s needs, provide emotional support and practical assistance, and foster a warm, inclusive family environment. On the other hand, clinicians can use family therapy to help patients and their families establish healthy communication patterns, strengthen family cohesion, and enhance support levels. By reinforcing family support, clinicians can effectively mitigate the negative effects of body image disturbances on social avoidance, helping patients recover psychologically and reintegrate into social life more quickly.

The relationship between family support and psychological resilience is complex, and clinical interventions should focus on both factors and their interactions. Clinicians can design intervention measures that combine family support and psychological resilience, such as family-participatory resilience training programs. These programs can help patients enhance their psychological resilience while receiving family support, enabling them to better address the challenges posed by body image disturbances. Burn patient rehabilitation is a long-term process, and the cultivation of psychological resilience and family support requires sustained attention and intervention. Clinicians can design phase-specific intervention plans based on the patient’s rehabilitation progress, gradually enhancing their psychological resilience and family support levels to promote comprehensive rehabilitation.

### 4.3 Operationalizing resilience and family support in burn rehabilitation

The present findings underscore that promoting psychological resilience and strengthening family support are not merely auxiliary goals but core components of comprehensive burn rehabilitation. To translate these insights into practice, resilience-building and family-integration strategies should be operationalized through three complementary levels.

Resilience-oriented care can be effectively integrated into existing rehabilitation workflows through the implementation of structured psychosocial modules. During the acute and subacute phases, nurses and clinical psychologists may administer brief resilience assessments (e.g., State–Trait Anxiety Inventory) at admission and discharge to identify patients who need targeted support. Evidence-based interventions, such as cognitive restructuring, mindfulness-based stress reduction, and goal-setting therapy, can be delivered as 15–20 minute guided sessions aligned with wound care or physiotherapy routines. Weekly interdisciplinary meetings should include a “psychological review,” allowing physicians, occupational therapists, and mental-health staff to jointly evaluate patients’ adaptive coping progress.

Health-care professionals require specific competencies to identify resilience deficits and reinforce adaptive coping behaviors. Training programs can be established using simulation-based workshops and case discussions focusing on communication skills, trauma-informed care principles, and motivational interviewing. These modules equip providers to recognize early signs of avoidance or maladaptive body-image concerns, respond empathically, and provide brief, structured psychological interventions at bedside. Integrating these competencies into continuing-education curricula or nursing residency programs will ensure sustainability and scalability.

Because family support significantly moderates the relationship between body-image disturbance and social avoidance, structured family inclusion is vital. Rehabilitation units can develop “Family Support Manuals” containing psychoeducation about scars, emotional responses, and effective reassurance techniques. Scheduled family participation sessions—for example, 30-minute weekly meetings moderated by a psychologist—can guide relatives to provide consistent positive feedback, encourage graded social re-exposure, and collaborate on home-based resilience exercises. Family members should also receive orientation on communication strategies that avoid overprotection or appearance-focused comments. Pilot studies in other trauma populations suggest that such structured engagement improves both patient self-efficacy and caregiver well-being, supporting the feasibility of adoption in burn care.

Institutions can formalize these practices through resilience-based clinical pathways that define care timelines, assign responsible staff, and establish outcome measures (e.g., changes in resilience scores, frequency of social engagement, and quality-of-life indices). Routine evaluation of these outcomes can inform quality-improvement initiatives and resource allocation. At a policy level, integrating resilience and family support training into national burn rehabilitation guidelines could facilitate broader uptake across the Chinese medical system, bridging the psychological and physical domains of recovery.

In summary, resilience enhancement and family participation should be conceptualized as operational, measurable, and team-driven processes. When systematically implemented, they can help interrupt the fear-avoidance cycle, accelerate psychosocial reintegration, and ultimately improve long-term recovery trajectories for burn survivors.

### 4.4 Limitations and future research directions

Despite the significance of this study, several limitations remain.

Although participants were recruited from two large tertiary hospitals that serve diverse populations across both urban and rural areas, the geographic concentration in Sichuan Province may still limit the generalizability of our findings to other cultural or healthcare contexts. Differences in family structures, medical insurance coverage, and rehabilitation resources across provinces or countries could influence the observed relationships among body image disturbance, psychological resilience, and social avoidance. Future studies should therefore adopt multi-center sampling across different socioeconomic and cultural regions, ideally incorporating national burn registry data to achieve broader representativeness and to test the cross-cultural invariance of the proposed moderated mediation model.

Second, the study used cross-sectional data, which, while confirming variable correlations, cannot establish causality between body image disturbances and social avoidance. For instance, long-term social avoidance may exacerbate body image disturbances by degrading social skills, further reinforcing negative self-perceptions. Additionally, the effects of psychological resilience and family support may vary dynamically across rehabilitation phases, but the current design did not capture such temporal effects, such as differences between the acute and recovery phases. Future studies can design prospective cohort studies with multiple assessments at 1, 6, and 12 months post-burn, establishing cross-lagged models to validate causal pathways. Combined with randomized controlled trials, researchers can test whether increased family support levels can directly improve psychological resilience and social avoidance. Concurrently, ecological momentary assessment data can be collected using mobile applications to record social avoidance behaviors and emotional fluctuations in real-time, thereby enhancing the explanatory power of the mechanisms involved.

Finally, another limitation concerns the reliance on self-report instruments, which may introduce biases such as social desirability bias, emotional recall bias, and common-method variance. To mitigate these effects, the study ensured complete anonymity during questionnaire administration, employed randomized item sequences, included reverse-coded items, and statistically examined potential common-method bias using Harman’s single-factor test, which indicated that no single factor accounted for the majority of variance. Additionally, all scales displayed high internal reliability, indicating consistent responses. Nevertheless, self-report methods cannot fully capture contextual or behavioral manifestations of social avoidance or resilience.

Furthermore, despite the inclusion of several clinical indicators (TBSA, burn stage, surgery status) to index injury severity, this study did not directly employ standardized objective assessments of scarring or appearance, such as the Vancouver Scar Scale. The absence of fine-grained scar visibility data may limit the precision with which self-perceived body image disturbance can be validated against observable disfigurement. Subsequent investigations should therefore adopt combined measurement models that integrate objective scar evaluations, photographic documentation, or clinician-rated indices with self-report tools. Such multimodal approaches will enable verification of whether subjective dissatisfaction accurately corresponds to measurable physical outcomes, thereby enhancing the ecological validity of body-image research among burn survivors. Furthermore, future research can collect data on the frequency and quality of support provided by family members. By using physiological indicators to quantify psychological stress or by exploring brain activation patterns in regions associated with body image using functional magnetic resonance imaging (fMRI), future studies can provide additional evidence on the psychological mechanisms underlying the mediating pathways.

## 5 Conclusion

This study established a novel regulatory mediation model within the fear-avoidance framework, revealing that body image disturbances in burn patients directly exacerbate social avoidance, while psychological resilience mediates this relationship by mitigating the negative effects of body image disturbances. Crucially, family support acts as a regulatory buffer, enhancing the protective role of resilience: high support protects psychological resources from being eroded by body image disturbances and promotes adaptive coping, whereas low support undermines resilience, even if it is initially present. These findings, which integrate family and resilience factors, extend the fear-avoidance model, suggesting that social avoidance stems not only from fear-driven avoidance cycles but also from modifiable interpersonal relationships and internal processes.

Clinically, this highlights the importance of integrating interventions that target individual resilience skills and family support systems. Rehabilitation programs should prioritize early screening based on burn severity, resilience-building modules, and caregiver involvement, particularly for high-risk groups, such as those with noticeable facial burns or economically disadvantaged families. Future efforts must explore mechanisms at a deeper level, cultural differences, and scalable implementation strategies to translate this evidence into equitable and effective psychosocial care for burn survivors worldwide.

## Data Availability

The raw data supporting the conclusions of this article will be made available by the authors, without undue reservation.
